# Life history traits and reproductive ecology of North American chorus frogs of the genus *Pseudacris* (Hylidae)

**DOI:** 10.1186/s12983-021-00425-w

**Published:** 2021-08-27

**Authors:** Jeffrey P. Ethier, Aurore Fayard, Peter Soroye, Daeun Choi, Marc J. Mazerolle, Vance L. Trudeau

**Affiliations:** 1 Department of Biology, University of Ottawa, Ottawa, ON K1N 6N5 Canada; 2 Centre d’etude de la Foret, Département des Sciences du Bois et de la Forêt, Université Laval, Quebec, QC G1V 0A6 Canada

**Keywords:** Chorus frogs, Life history, Distribution, Conservation, Population management

## Abstract

Amphibian biodiversity is declining globally, with over 40% of species being considered threatened to become extinct. Crucial to the success of conservation initiatives are a comprehensive understanding of life history and reproductive ecology of target species. Here we provide an overview of the *Pseudacris* genus, including breeding behaviour, reproduction, development, survival and longevity. We present an updated distribution map of the 18 species found throughout North America. We also summarize the conservation status at the national and subnational (state, provincial, and territorial) levels, in Canada, USA, and Mexico, to evaluate the relationship between life history traits and extinction risk. Results show a high degree of consistency in the life history traits of *Pseudacris* species considering their relative diversity and wide distribution in North America. However, data are lacking for several species, particularly in the Fat Frog and West Coast clades, causing some uncertainties and discrepancies in the literature. We also found that the most threatened populations of chorus frog were located in the east coast of the USA, potentially as a result of increased levels of anthropogenic disturbance. We suggest that the similarities in life history traits among chorus frog species provides an opportunity for collaboration and united efforts for the conservation of the genus.

**Supplementary Information:**

The online version contains supplementary material available at 10.1186/s12983-021-00425-w.

## Introduction

The biodiversity of wildlife is declining globally [1, 2]. These declines are related to several factors including habitat destruction, introduced pathogens, and climate change [1, 2], and are associated with a loss of ecosystem function [3, 4]. Amphibian species appear to be affected disproportionately to other taxa [5, 6] with over 40% of amphibians considered threatened to become extinct worldwide [6–9]. North America is no exception, as amphibian species have experienced drastic declines since the 1960s [10]. In the USA, 56 amphibian species are threatened to become extirpated [2], and the average rate of decline of local amphibian populations is almost 4% annually [10]. In Canada, 22 amphibian species are listed as “endangered”, “threatened”, or of “special concern” [11]. Mexico supports the greatest number of amphibian species in North America (~ 372 species), many of which are endemic [12, 13]. According to Pasquali [14], 220 amphibian species (~ 60%) are considered at risk of extinction in Mexico. These declines are concerning because amphibians have life stages in both aquatic and terrestrial habitats and hold an important ecological role through supporting services for primary production, decomposition, and nutrient cycling [15, 16]. Amphibians also act as bioindicators that provide an early warning system to degradations in ecosystem health and environmental change [7, 8, 17].

Conservation actions such as captive breeding and reintroductions have been initiated to maintain some wild populations, while other efforts are taken to mitigate the sources of population declines [18, 19]. The Amphibian Conservation Action Plan was created in 2007 with the goal to preserve amphibian biodiversity worldwide by providing an overview on how to expand knowledge, monitor and document diversity, and respond to threats to amphibian species and their habitats [20, 21]. Since the Amphibian Conservation Action Plan has been established, amphibian reproduction ex situ in zoos has been prioritized in many regions [19]. Specimens are collected and kept in captivity to maintain the genetic diversity of extant populations and to increase population abundance through captive breeding or translocation to new or historical habitats [22]. Amphibians are often good candidates for captive breeding because they tend to have higher fecundity, smaller body size, and lower associated costs for husbandry compared to other taxa [18, 23]. Captive populations are currently maintained in zoos and academic institutions for several North American amphibian species, including northern leopard frogs (*Lithobates pipiens*) [24, 25], dusky gopher frogs (*Lithobates sevosus*) [26, 27], Wyoming toads (*Anaxyrus baxteri*) [28, 29], axolotls (*Ambystoma mexicanum*) [30] and hellbenders (*Cryptobranchus alleganiensis*) [31], amongst others.

Gathering knowledge on natural population dynamics is a crucial initial step before conducting a captive breeding program, because the evaluation of success will be based on parameter values in wild populations. Although every aspect of population ecology has potential to inform recovery strategies, reintroduction success is often evaluated using indicators such as survival rates, demography, and fecundity [32]. Therefore, a comprehensive understanding of the life history traits of species of interest is essential for their recovery. This information is ideally gathered when species are abundant or when population declines are first detected before species become imperilled.

Chorus frogs (genus *Pseudacris*: Hylidae) are an example of a clade that is relatively abundant but with several populations that have experienced significant declines [33–36]. This species group occurs in North America and is distributed widely across Canada, the United States and Mexico. These frogs are of cultural significance, as a symbol of fertility and renewal [37] and a source of food for indigenous peoples of North Americans [38]. The call of groups of male chorus frogs is a familiar sound of spring for people living in suburban areas [39]. Curiously, the frog calls heard in many movies and television shows as ambient noise in nighttime scenes is that of the Pacific chorus frog (*P. regilla*) [40]. Chorus frogs have also been used as flagship species, representing conservation initiatives. For example, the boreal chorus frog (*P. maculata*) is a symbol for the protection of threatened species in Québec, Canada [41]. Chorus frogs play an important role in North American food webs. Larvae consume algae and adults consume insects, while chorus frogs are prey items for birds, fishes, and other animals, thus cycling nutrients between aquatic and terrestrial ecosystems [42]. Despite the significance of chorus frogs, there is very limited knowledge on the physiology and ecology of several species. The majority of information for many of the chorus frog species was collected in the first half of the twentieth century and requires updating. Significantly, revisions in the nomenclature and phylogenetic assignment make historical accounts confusing and challenging to interpret [43, 44], prompting this review of existing information.

Our objectives are to summarize the ecology, life history strategies, and conservation status of North American chorus frogs. First, we present a map of the distribution of the 18 species using the most up to date taxonomic classifications. Second, we present a summary of the general ecology of these species, with a focus on breeding behaviour, reproduction, and development. Third, we review the life history strategies of chorus frogs. We searched databases (Web of Science and Google Scholar) for articles pertaining to chorus frog species. We highlight the differences among species and taxonomic clades as well as gaps in current knowledge. We also compare the current conservation status of chorus frog species to explore if patterns of distribution and reproductive strategies are associated with extinction risk.

## Methods

### Taxonomic note

**Fig. 1:**
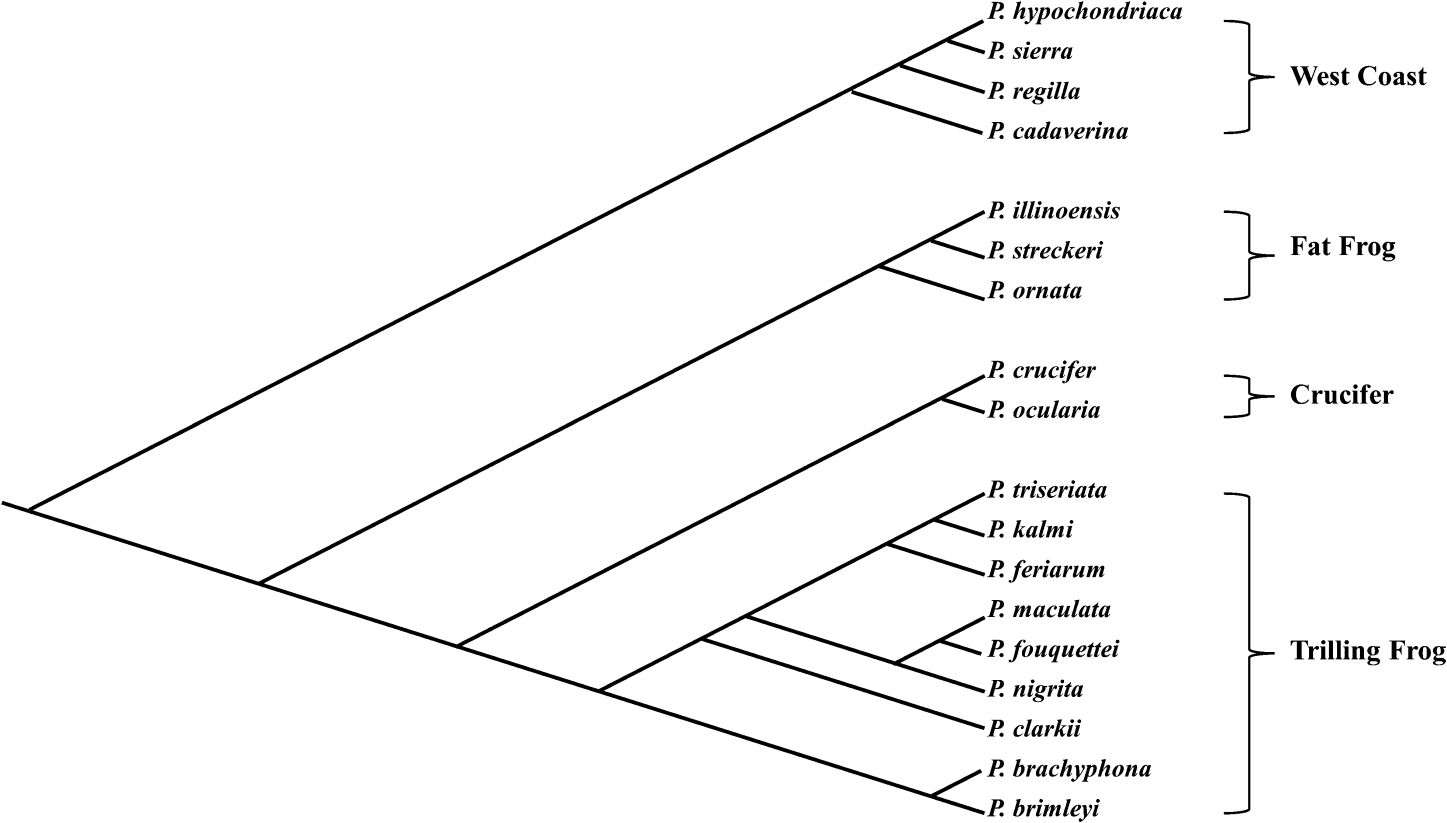
Cladogram of 18 species of chorus frog (genus *Pseudacris*: Hylidae). Branching is based on the phylogeny estimated with *BEAST using the multiple-allele dataset (26 nDNA loci) published by Barrow et al. [44]. Note that the branch lengths delineate estimated relatedness but do not represent the evolutionary time between two nodes

The taxonomy of the members in the genus *Pseudacris* is widely debated. The nomenclature and species status of many animals within this genus has changed repeatedly over the last 70–80 years [45–48]. As such, it is often difficult to determine which species is being described in studies published throughout this time period. To avoid confusion, some authors group closely related species or subspecies into species complexes (i.e., *Pseudacris triseriata* complex) or reinstate historical classification in separate genera (i.e., *Hyliola* for *P. regilla* and *P. cadaverina*) [49–51]. Others have split species concepts based on geographic distribution [48]. However, recent advancements in genetic sequencing have yielded some insight into this problem with taxonomic classification. Studies from the past 20 years [43, 44, 52, 53] indicate that there are at least 16 species within the genus, which can be separated into four clades of related species: (1) the West Coast clade containing *P. regilla* and *P. cadaverina*, (2) the Fat Frog clade containing *P. ornata*, *P. streckeri*, and *P. illinoensis*, (3) the Crucifer clade containing *P. crucifer* and *P. ocularis*, and (4) the Trilling Frog clade containing *P. brimleyi*, *P. brachyphona*, *P. clarkii*, *P. feriarum*, *P. fouquettei*, *P. kalmi*, *P. maculata*, *P. nigrita*, and *P. triseriata*. These distinctions are based on a combination of nuclear and mitochondrial DNA analyses, and morphological and behavioural data. More recent genetic studies and updates to nomenclature have listed two additional species in the West Coast clade, which are closely related to *P. regilla*; the Sierran chorus frog (*P. sierra*) and the Baja California chorus frog (*P. hypochondriaca*) [51, 54, 55]. However, these nomenclatural updates are still debated [44] and information regarding life history and reproductive ecology is very limited for these two species. Some authors favour the re-establishment of the genus *Hyliola* for the species within the West Coast clade based on geographic separation from the other species [51]. Finally, recent genetic, acoustic, and ecological research on *P. brachyphona* by Ospina et al. [56] suggests that northern and southern populations in this species are distinct. Ospina et al. [56] propose that the southern populations be considered as a separate species, the Collinses’ Mountain Chorus Frog (*P. collinsorum*). For the purpose of this review, we have chosen to retain the *Pseudacris* nomenclature, and include *P. sierra* and *P. hypochondriaca* in the West Coast clade, but not include *P. collinsorum* in the Trilling Frog clade given it is not currently recognized by the Society for the Study of Amphibians and Reptiles [57]*.* We will discuss these 18 species (Fig. 1, Additional file 1: Table S1) as they are described by Barrow et al. [44], and use descriptions of species’ distributions to determine the likely identity of the species when considering articles published prior to 2010s.

### Mapping distributions

To map the distributions of the 18 species of North American chorus frog, we downloaded all *Pseudacris* occurrence data recorded on the basis of preserved specimens, material samples, and human or machine observation from the Global Biodiversity Information Facility (GBIF; [58]). We removed data that did not contain spatial coordinates or information on the year they were recorded. We also removed records that were not identified to species, and records where the GBIF indicated the spatial coordinate uncertainty was potentially invalid. We also cross-referenced all occurrence points for species with their known distributions according to experts (i.e., Tables 1 and 3). Recent genetic studies revealed that the distribution of *P. triseriata* in Canada is largely confined to southern Ontario [43, 44]. Populations of chorus frogs north of Wellington County (Ontario), which were previously believed to be *P. triseriata*, are now known to be *P. maculata*. Therefore, we excluded any *P. triseriata* observations in Canada north of 44 degrees latitude. This resulted in a dataset of 72,199 species observations, collected between 1812 and 2021. For each species, we created concave hull polygons around the occurrence records, and created buffers around these polygons equivalent to the highest recorded measure of coordinate uncertainty for that species, to a maximum of 100 km. These final polygons represent the best-known distribution of each species according to all available occurrence data. All data were processed using R (version 3.6.1), using the packages tidyverse [59], raster [60], and rgbif [61]. Mapping and visualization of spatial data was done in ArcGIS Pro (version 2.5.1).

**Table 1:** Summary of life history traits of *Pseudacris* species in North America, separated by clade. See Additional file 3: Table S3 for list of abbreviations

Clade	Species	Distribution	Breeding season	No. of eggs	No. eggs per cluster	Time to hatch	Time to metamorphose	References
West coast	*P. cadaverina*	**USA**: CA **MEX**: BCN	Feb–Oct	–	1–2 eggs	–	40–75 days	[50]
	*P. hypochondriaca*	**USA**: AZ, CA, NV, UT **MEX**: BCN, BCS	Nov–July	400–750	9–80 eggs	2–9 days	60–75 days	[50, 62, 63]
	*P. sierra*	**USA**: CA, ID, MT, NV, OR, UT	Nov–July	400–750	9–80 eggs	–	60–65 days	[50, 62, 64]
	*P. regilla*	**USA**: AK, CA, MT, OR, WA **CAN**: BC	Nov–July	400–750	9–80 eggs	7–21 days	52–70 days	[65, 65–71]
Fat frog	*P. illinoensis*	**USA**: AR, IL, MO	Feb–March	200–1000	8–79 eggs	–	–	[72–76]
	*P. ornata*	**USA**: AL, FL, GA, LA, MS, NC, SC	Nov–March	10–106	20–40	~ 7 days	~ 90 days	[77–79]
	*P. streckeri*	**USA**: AR, IL, KS, LA, MO, OK, TX	Nov–March	≤ 600	–	2–5 days	~ 60 days	[65, 80, 81]
Crucifer	*P. crucifer*	**USA**: AL, AR, CT, DE, FL, GA, IA, IL, IN, KS, KY, LA, MA, MD, ME, MI, MN, MO, MS, NC, NH, NJ, NY, NY, OH, OK, PA, RI, SC, TN, TX, VA, VT, WI, WV **CAN**: MB, NB, NL, NS, ON, PE, QC	Nov–June	~ 700	2–3 eggs	6–15 days	~ 90 days	[65, 82–87]
	*P. ocularis*	**USA**: AL, FL, GA, NC, SC, VA	Jan–Sept	≤ 200	1–25 eggs	1–2 days	7–70 days	[65, 88, 85, 89]
Trilling frog	*P. brachyphona*	**USA**: KY, MD, OH, PA, TN, VA, WV	Feb–June	300–1500	4–144 eggs	7–10 days	30–64 days	[90, 90–93]
	*P. brimleyi*	**USA**: GA, NC, SC, VA	Feb–April	≤ 300	–	–	30–60 days	[94]
	*P. clarkii*	**USA**: KS, OK, TX **MEX**: TLA	Jan–June	~ 1000	3–60 eggs	2–3 days	30–45 days	[65, 95–97]
	*P. feriarum*	**USA**: AL, DC, FL, GA, IL, KY, MD, MO, MS, NC, NJ, PA, SC, TN, VA, WV	Feb–May	≤ 1000	40–60 eggs	7–14 days	40–90 days	[86, 98]
	*P. fouquettei*	**USA**: AR, LA, MO, MS, OK, TX	Jan–May	500–1500	–	2–3 days	–	[53, 99–102]
	*P. kalmi*	**USA**: DE, MD, NJ, PA, VA	Feb–April	500–1500	6–20 eggs	7–14 days	40–90 days	[103, 104]
	*P. maculata*	**USA**: AZ, CO, IA, ID, IL, IN, KS, MI, MN, MO, MT, ND, NE, NM, NY, OK, SD, UT, VT, WI, WY **CAN**: AB, BC, MB, NT, ON, QC, SK, YK	Feb–April	137–793	5–100 eggs	10–14 days	~ 60 days	[99, 105–108]
	*P. nigrita*	**USA**: AL, FL, GA, LA, MS, NC, SC, VA	Dec–Sept	≤ 180	6–176 eggs	2–3 days	40–120 days	[65, 77, 109, 110, 109–112]
	*P. triseriata*	**USA**: IL, IN, KY, MI, NY, OH, PA **CAN**: ON	Jan–June	440–1500	20–70 eggs	3–27 days	40–90 days	[65, 113–115]

2-Letter state/province codes for USA and Canada, and 3-letter state codes for Mexico as per ISO 3166-2 (i.e., *BCN* Baja California, *BCS* Baja California Sur, *TLA* Tlaxcala). Region abbreviation (i.e., USA, CAN, MEX) are bolded for legibility and hold no other significance

### Life history literature review

We compiled empirical data on the life cycle and population dynamics of the 18 *Pseudacris* species of the genus [43, 44, 52, 116], considering all life stages, from the egg to the adult stage. We employed advanced searches with keywords in Web of Science and Google Scholar (Additional file 2: Table S2) performed between October 2020 and January 2021. We selected these reference databases as they produced more results (number of articles, books, and dissertations included), compared to BioOne, BioRxiv and Science Direct. We did not restrict results by languages or period. After reading the abstract, we retained relevant articles, books, and dissertations that dealt with the survival and the reproductive cycle of wild populations or from lab experiments of the target group. We extracted information pertaining to geographic distribution, breeding season length, fecundity (total number of eggs, number of eggs per cluster), development (time to eggs hatching, time to metamorphosis), stage-specific survival (eggs, larvae, juveniles, adults), age of maturity, and longevity. We excluded any document that did not clearly present an estimate (i.e., count, proportion, percentage) in the main body of the text, including tables and figures. When estimates of a given parameter were found in several sources, we presented the range of values. We did not distinguish between data obtained under controlled conditions (mesocosm or laboratory) and data from observational field studies, or between different methodologies of data collection.

### Conservation status

Global status and population trends were assessed using the Red List of Threatened Species database of the International Union for Conservation of Nature [2]. To determine the national and subnational (i.e., provincial or state level) conservation status and distribution of the *Pseudacris* species, we utilized the NatureServe Explorer database [117]. Status ranks are given the prefix code “N” for national status and “S” for subnational status, and a numerical suffix ordered from 1 (critically imperilled) to 5 (stable). Combinations of codes can be used to indicate uncertainty, such as S2S3 representing a status being either imperilled or vulnerable. There are also a series of unique codes (i.e., SH = possibly extinct at the subnational level, SNR = unranked or not assessed at the subnational level). See Additional file 4 for a full explanation of status ranks and a list of abbreviations (Additional file 3: Table S3). NatureServe databases primarily contain conservation status and distribution information in the United States of America, Canada, and Latin America. For species that are known to be extant in Mexico, we supplemented the distribution data with information from the USGS Nonindigenous Aquatic Species database [118] and AmphibiaWeb [119]. Ranking was then compared to the conversation status as stated by the threatened species legislation of each North American country: Canada (Species at Risk Act, S.C. 2002, c. 29), United States of America (US Endangered Species Act, 1973, 16 U.S.C.), and Mexico (Norma Oficial Mexicana, NOM-059-ECOL-2001, Secretaría de Medio Ambiente y Recursos Naturales 2002).

## Results

Our initial search of life history data using Web of Science and Google Scholar produced a total of 15,464 results, and we retained a total of 109 documents published between 1924 and 2020 for our review. Of these, over two-thirds (67.9%) were published prior to 2000, and nearly a third (31.2%) were published prior to 1970. The most widely distributed *Pseudacris* species were the most represented in the search; namely *P. crucifer*, *P. maculata*, *P. triseriata*, and the three species originally classified as *P. regilla*. The majority of the information was collected from regions along the coast of eastern USA (24.8% of documents; Florida, Georgia, Maryland, Pennsylvania, New York, New Jersey, North Carolina, South Carolina), in midwestern USA (18.3%; Illinois, Iowa, Michigan, Minnesota, Kansas, Wisconsin), and in California (11.0%). Only two documents (1.8%) featured data from species that occur in Mexico, and five (4.6%) from species in Canada.

### Distribution map

**Fig. 2:**
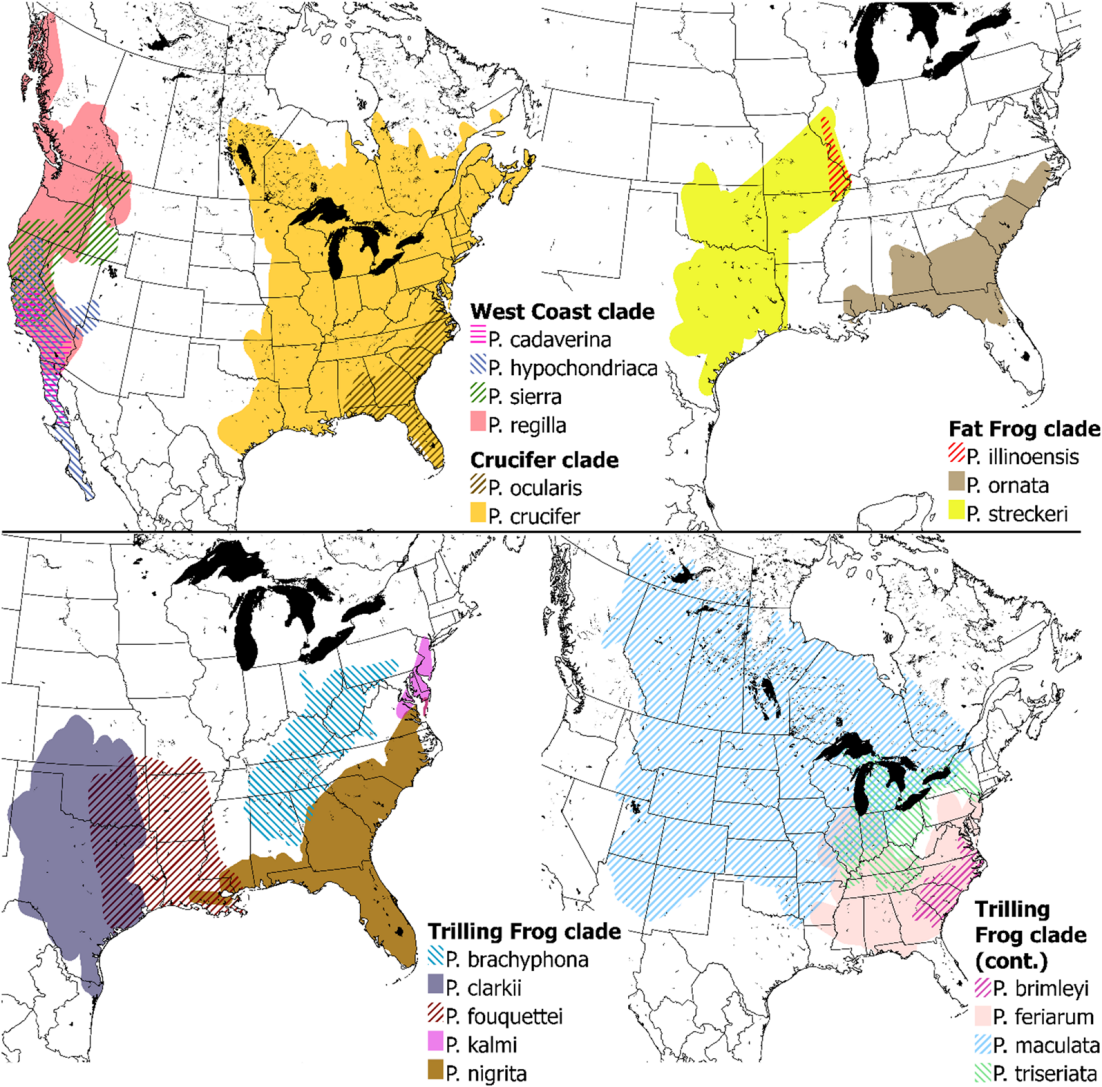
Distribution of the 18 species of North American chorus frogs (genus *Pseudacris*: Hylidae), separated by phylogenetic clade. Distribution is based on occurrence data from the Global Biodiversity Information Facility website [58]

Chorus frogs are found throughout Canada, USA and the Baja California peninsula of Mexico (Fig. 2). West Coast clade frogs are distributed throughout the Pacific coast and western Canada and USA, including British Columbia, Alberta, Washington, Oregon, California, Nevada, and Arizona. Within Crucifer clade frogs, *P. crucifer* is widespread throughout eastern Canada and USA, whereas *P. ocularis* is confined to the southeastern coast of the USA, in Virginia, Georgia, North Carolina, South Carolina, and Florida. Species from the Fat Frog clade are found across central to southern USA, as far north as Illinois (*P. illinoensis*), west throughout Oklahoma, Missouri and eastern portions of Texas (*P. streckeri*), and south and eastward into northern Florida and the southeastern seaboard. The Trilling Frog clade is the most extensively distributed. In particular, *P. maculata* are a widespread species found throughout central Canada and northeastern USA, from the Northwest Territories in the north, British Columbia, Idaho, Utah and Arizona in the west, to Quebec, Michigan, Indiana, Missouri, and Louisiana in the east. *P. nigrita* and *P. feriarum* occur in the southern and northern portions of the east coast of the USA, respectively. In the southwest of the USA, *P. clarkii* are found in Texas, Oklahoma, Kansas and Tlaxcala (Mexico), whereas *P. fouquettei* are found in Texas, Oklahoma, Alabama, Arkansas, and Mississippi. The most restricted species within the Trilling Frog clade is *P. kalmi*, which are found in small portions of Delaware, Maryland, New Jersey, Pennsylvania, and Virginia.

### General ecology and life history traits

#### Morphology

**Fig. 3:**
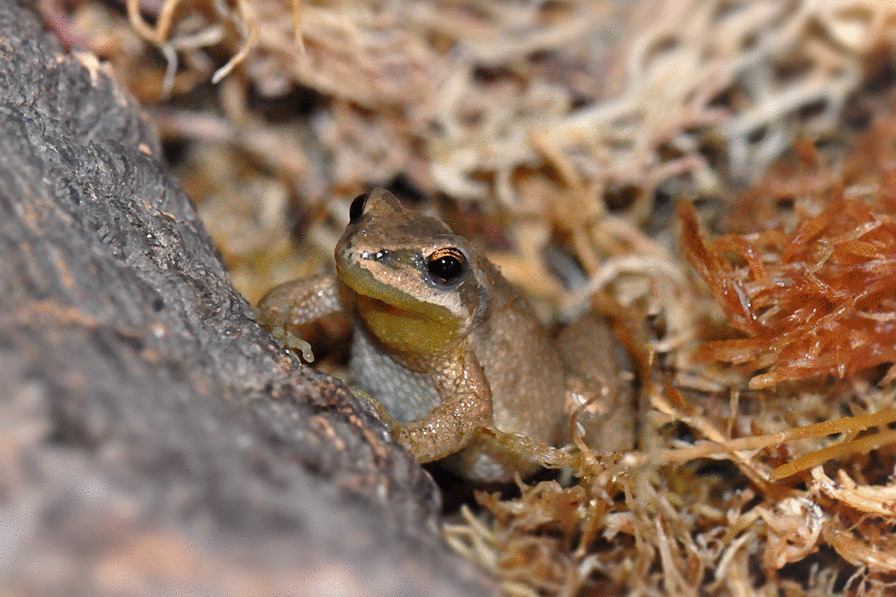
Adult male boreal chorus frog (*Pseudacris maculata*), Great Lakes/St. Lawrence—Canadian Shield population, reared in captivity. Age = 10 months. Snout-vent length = 29.9 mm. Photo by J.P. Ethier (04 March 2021)

Chorus frogs are small-bodied (approximately 2–4 cm, 1–5 g as adults), often heard but rarely seen [41, 49]. They tend to be slender with a slim waist and long limbs (Fig. 3). Toe discs are small with minimal webbing between digits [49, 65, 99]. Most species have a light line on the upper lip [49, 65, 99]. Males possess a single, round vocal patch, which is yellow, grey or brown over a lighter background colour. Bellies tend to be free of pigmentation. Both sexes often have dorsal patterns with rows of dark spots, stripes, or a cross (“X”) over a brown, green, or cream body coloration. However, coloration may be highly variable, even within populations of the same species [99, 120]. Some species, such as *P. regilla* and *P. sierra*, may be able to change colour within a season [120–122]. Albinism (lack of pigment) and erythrism (red pigmentation) have also been recorded in chorus frogs [113, 123, 124]. Lemmon et al. [53] provide an excellent comparison of morphology among several species in the Trilling Frog clade.

#### Timing of breeding

Like most anurans, chorus frogs are described as polygynous, or “lekking” species [125, 126]. Chorus frogs are also iteroparous, although mortality is very high in the first year, so many individuals only participate in a single breeding season during their lifetime [77, 105, 127]. More recent studies suggest that the proportion of chorus frog that breed more than once is greater than previously thought [128, 129]. Thus, long-term studies are required to evaluate the contribution of individuals to the reproductive effort across several breeding seasons. Although most chorus frogs reach sexual maturity by the end of the first summer, individuals generally do not breed during the first year [113]. Species in this genus apparently capitalize on “cold weather breeding” in late winter and early spring to avoid competition with other hylid frogs [52]. The timing of reproduction and calling behaviour is influenced by rainfall and temperature [95, 100, 130]. However, environmental variables are not the sole determinants of reproduction and calling behaviour, as indicated by the asynchrony of timing of reproduction of sympatric species [65, 77, 113, 126, 131]. Breeding seasons are highly variable (Table 1), generally reaching its peak in March–April in eastern regions, December–February in southern and western regions, and can occur over a prolonged period [95, 125, 126, 132]. For example, several species with southerly distributions can be observed breeding almost year-round, beginning as early as October and extending into the summer of the following year (*P. cadaverina*: [133, 134]; *P. ocularis*: [88]; *P. ornata*: [65]; *P. nigrita*: [109, 110]; *P. regilla*: [66, 135]; *P. streckeri*: [131, 136, 137]).

At the beginning of the breeding season, males gather in large groups shortly after emerging from hibernation, and remain within the breeding habitat for 4–10 weeks [77, 113, 138]. Conversely, females are often present in the breeding habitat for only a few nights for up to 2 weeks [77, 113]. Sex ratios on breeding grounds are generally biased towards males [36, 77, 139, 140]. After spawning concludes, males will continue to call to attract more mates, whereas females will return to terrestrial habitat after oviposition. Both males and females are capable of mating with multiple individuals, but for the majority of species usually only one clutch of eggs will be produced per breeding season [126, 141]. However, multiple clutches have been observed in *P. triseriata* [142], and *P. regilla* (and potentially *P. sierra* and *P. hypochondriaca*) may produce as many as three egg clutches in a season [62]. Recently, Goldberg [132] reported that chorus frogs can spawn twice in the same breeding season. Indeed, female *P. streckeri* specimens collected in Oklahoma had both mature and post-ovulatory follicles in the same ovary, indicating multiple spawning events within a single breeding season [132].

#### Breeding habitat

Reproduction is aquatic in all species in the *Pseudacris* genus. A wide variety of shallow water habitats, both natural and artificial, are utilized for breeding [99]. The majority of species use temporary or semi-permanent water bodies that are relatively free of predators and heterospecific competitors [143, 144]. Breeding habitats include temporary ponds, roadside ditches, flooded meadows, shallow bogs and marshes, buffalo wallows, furrows in plowed fields, glacial kettlepots, as well as ephemeral pools and vernal pools in woodlands [49, 65, 113, 145, 146]. Most breeding sites are lentic freshwater systems, but Pacific chorus frogs (*P. regilla*) and California tree frogs (*P. cadavernia*) also breed in small, slow-moving streams [65, 133, 134].

#### Calling behaviour

Reproductive behaviour is initiated by males vocalizing, and long periods of calling likely have a role on circulating reproductive hormone concentrations and in maintaining sexual arousal in females [138, 147]. Chorus frogs get their common name from their calling behaviour [65, 148]. When a sufficient number of males have gathered and are calling, a chorus of near continuously calling individuals is established [138, 149]. These aggregations of calling males allow females to assess the relative quality of potential mates, and for males to assess the quality and competitive abilities of other males [138]. In several species (i.e., *P. crucifer, P. regilla, P. triseriata*), males produce a variety of calls, including advertisement calls and courtship calls [138, 150]. Advertisement calls are long-range vocalizations that signal the position of a male to other males, and to attract females. Courtship calls are short-range vocalizations produced by males that are directed towards nearby females to indicate an “eagerness” to mate [138, 150].

Male *Pseudacris* frogs produce either a series of repeated single notes, whistles, or a long trill [138]. The advertisement call of male boreal chorus frogs (*P. maculata*) is described as a series of pulses, 750–905 ms in duration, produced at a rate of approximately 16 pulses s^−1^ [151]. Calls are similar among species within the Trilling Frog clade, but see Cocroft and Ryan [152] for comparisons of temporal and spectral properties of calls among species within the Trilling Frog clade. The call of *P. brachyphona* is more rapid and high pitched, and described as “quack like” rather than a trill [153]. Within the West Coast clade, calls consist of a one- or two-phase “rib-bit”, which contains a series of pulses, approximately 232–245 ms in duration, delivered at a rate of 86–90 pulses s^−1^ [154]. Species in the Fat Frog clade produce very short whistles (30–60 ms) repeated in quick succession (*P. ornata* and *P. streckeri*) [136].

Pulse rate and call duration are important properties for species recognition in mixed species assemblages [141, 151, 155]. However, there is plasticity in calling behaviour, with the properties of calls influenced by environmental conditions, temperature being predominant [138, 152, 156]. Patterns in acoustic signals are also influenced by the social context. For example, male spring peepers (*P. crucifer*) produce tone-like “peeps” when calling in a chorus (advertisement call), but produce trill-like calls of short pulses (aggressive call) when in close proximity to a competing male [157]. Males also increase the duration and intensity of their advertisement calls as the spacing between males decreases [158]. Similar patterns have been noted in other *Pseudacris* species [159].

Some male individuals may adopt a non-calling strategy [160]. These silent males are often referred to as satellites and associate closely with a calling male [138]. Unlike in other anuran species, this behaviour is apparently not size specific nor associate with “inferior” males that cannot effectively compete [161]. Individual males may switch between the calling or non-calling strategy within a single night [142, 162–164]. Presumably, this strategy is used to intercept females as they approach a calling male [165]. However, an alternative hypothesis is that these males remain silent to conserve energy while waiting for calling territories to become available [142].

#### Amplexus

Consistent with other genera in the Hylidae family, *Pseudacris* species perform axillary amplexus. The male mounts the female, grasps her directly behind the forelimbs, with the male cloaca positioned above the female cloaca [126, 138, 166]. This behaviour is initiated by female contact, indicating receptivity. Males that attempt to mount an unreceptive female are quickly dissuaded by the female moving away, although this avoidance behaviour is not always successful [111, 142]. Amplexus usually only occurs at night, but *P. kalmi* have been observed in amplexus during the day [103]. Observations of *P. crucifer* [82] and *P. triseriata* [103] suggests that ovulation precedes amplexus. Mates remain in amplexus between a few hours up to 40 h, as observed in *P. regilla* [167]. Amplexus behaviour is concurrent with oviposition. Prior to oviposition, *P. nigrita* females perform “spasmodic” abdominal contractions [111].

#### Oviposition

As the *Pseudacris* female releases her eggs, she will arch her back bringing her cloaca in close proximity to the male cloaca [111, 147]. For the majority of species, this behavior occurs as the female straddles some form of submerged vegetation to which the eggs are attached [90, 111, 113]. The duration of oviposition is variable and often occurs in several successive events over the course of 2–3 h, with the female and male in amplexus moving between locations [99, 111, 138]. Eggs are laid singly or in small clusters, depending on the species (Table 1). Whitaker [113] noted that egg-laying in *P. triseriata* occurred at temperatures > 10 °C, and often after rainfall. Clutch size, or the full complement of eggs deposited as one to several masses, is relatively small in comparison to related taxa, such as treefrogs in the genus *Dryophytes* (= *Hyla*) that can have clutches of 2000–4000 eggs [168]. For example, Southern chorus frogs (*P. nigrita*) lay ≤ 160 eggs in a series of masses of approximately 15 eggs [111, 112]. At the other extreme, several species of the former “*P. triseriata* complex” including *P*. *feriarum*, *P*. *kalmi*, *P*. *maculata*, and *P. triseriata* deposit up to 1500 eggs in masses of approximately 10–80 eggs [146]. Similarly, large clutches (1479 eggs) have also been observed in *P. brachyphona* [91]. Oviposition behaviour can be altered in response to predators and competition [169–171]. Buxton et al. [172] found that female *P. triseriata* lay fewer eggs in experimental ponds that contained western mosquitofish (*Gambusia affinis*) than females in ponds that were fish-free. Ouellet et al. [143] observed that *P. maculata* breeding sites in Québec (Canada) were generally devoid of predatory fish. Reproductive investment and fecundity are associated with body size in several frog species, including those in the families Hylidae, Leptodactylidae, Microhylidae, Ranidae and Rhacophoridae [173–176]. Duffitt and Finkler [177] found that, prior to reproduction, larger males and females of *P. crucifer* and *P. triseriata* allocate more energy to courtship activity and gamete production, respectively, than smaller individuals. Ovarian mass is positively correlated with body size in both species, and the gonadal-somatic index is positively correlated with body size in *P. crucifer* [177].

#### Development

Eggs generally hatch within 2 weeks of being deposited, but can range from 2 to 27 days [146, 178]. As with many amphibian species, egg and tadpole development depends on water temperature, hydroperiod and other environmental conditions [113, 179–181]. At metamorphic emergence, *P. brachyphona* and *P. crucifer* have a balanced sex ratio [82, 91]. Larvae are generalist feeders, indiscriminately consuming a variety of items including detritus, algae, and other periphyton associated with submerged vegetation [146, 178], as well as small quantities of pollen and invertebrates [182–184]. The larval period is short in most species, with metamorphosis (Gosner stage 46) occurring 30–90 days after hatching [146]. To assess the influence of hydroperiod on tadpole development, Amburgey et al. [181] collected boreal chorus frog (*P. maculata*) tadpoles from permanent and temporary ponds (Gosner stage 24–31), and then subjected tadpoles to one of three hydroperiod regimes. Whereas the hydroperiod treatment did not influence development rate, tadpoles collected from permanent ponds matured and metamorphosed faster than those collected from temporary ponds. The authors hypothesized that developmental rates are influenced by predation level as a wider variety of predators are more likely to be found in larger and more permanent water bodies [181].

#### Migration and hibernation

After reaching metamorphosis, juvenile frogs remain near natal ponds for several weeks and then migrate a short distance (< 500 m) into more terrestrial habitats close to water [50, 99, 105]. Migration distance varies between populations and depends on the distribution of suitable habitats [185, 186]. The majority of pond-breeding amphibians are highly philopatric [186]. Since most *Pseudacris* species utilize temporary bodies of water, individuals may be philopatric to a general area rather than a specific water body and regularly switch ponds. This pattern is especially common in regions where stochastic environmental or anthropogenic conditions result in ponds regularly being created or drying up [186, 187]. Juvenile habitat is largely similar to adult habitat, but has not been extensively studied in any species [50].

Based on observations of *P. clarkii*, *P. crucifer*, and *P. ocularis,* adult chorus frogs are primarily terrestrial, only found in aquatic environments during breeding, and will migrate short distances away from ponds and pools after spawning [96, 188]. Adults generally remain within 100 m of breeding ponds during the spring and summer, and rarely migrate > 200 m within a single generation (*P. triseriata*: [189]; Trilling Frog clade: [116]). Conversely, Green [91] observed migrations of up to 610 m within a single breeding season and up to 1219 m between breeding seasons in mountain chorus frogs (*P. brachyphona*).

Most populations of chorus frogs enter torpor and overwinter in terrestrial habitats, either underground or under logs, rocks, and leaf litter [50, 92, 190]. Chorus frogs may migrate short distances to hibernation sites but are generally found emerging from locations close to breeding sites [113, 189]. Spring peepers (*P. crucifer*), Pacific tree frog (*P. regilla*), Western chorus frogs (*P. triseriata*), and boreal chorus frogs (*P. maculata*) tolerate temperatures below 0 °C. These species produce a glucose-based cryoprotectant limiting cell volume reduction and preventing intracellular freezing during sub-zero temperatures [191–197]. It is unclear whether *Pseudacris* species with southern distributions have the ability to utilize similar freeze tolerance or freeze avoidance mechanisms. Indeed, not all species are thought to hibernate. Some populations of the ornate chorus frog (*P. ornata*) and little grass frog (*P. ocularis*) are active during the winter months and may even breed during this time [88, 198, 199].

#### Stage-specific survival probability

##### Eggs and larvae

Mean survival probability was highly variable among species and published studies (Table 2). The majority of data on egg and larval survival probability have been collected with species in the Trilling Frog clade, and we did not find any survival estimates on several species, including *P. cadaverina, P. brachyphona* and *P. brimleyi.* Development and survival probabilities in the aquatic stages depend on several abiotic and biotic factors, such as predation and competition rates, hydroperiod, and water quality [181, 200]. In general, survival probabilities are higher in controlled settings compared to natural conditions as eggs and larvae are able to develop without predation pressures and risks of desiccation, and with more stable environmental conditions [201]. In a natural population, Whiting [105] reported a mean survival probability of only 0.05 (*P. maculata*). Most studies reviewed measured the hatching success by transferring eggs or larvae into a controlled environment [90, 114, 202–204]. Even when major threats are eliminated in controlled environments, *Pseudacris* species can experience high rates of mortality between hatching and the end of the larval period. For example, survival probability of eggs was estimated to be 0.39 for *P. clarkii* [204]. Survival probabilities can also be relatively high in natural settings. In *P. triseriata* reared in natural ponds, Kramer [205] reported a mean survival probability of approximately 0.62 for eggs, and Smith [206] reported a survival probability from larvae to metamorphosis between 0.25 and 0.90. Due to the lack of data, comparisons among species and clades during the aquatic stages are very limited.

**Table 2:** Summary of survival probabilities (φ) and longevity in frog species in the genus *Pseudacris*, separated by clade

Clade	Species	φ eggs	φ larvae	φ juveniles	φ adults	Lifespan	Age at maturity	References
West coast	*P. cadaverina*	–	–	–	–	–	–	–
	*P. hypochondriaca*	0.85–0.95	–	–	0.01–0.3	–	1 year	[66, 207]
	*P. sierra*	–	0.90–0.95	–	–	–	–	[64]
	*P. regilla*	–	–	–	–	1–3 years	1–3 year	[63, 208, 209]
Fat frog	*P. illinoensis*	–	–	0.03–0.04	0.28	2–6 years	1 year	[36, 210, 211, 210–212]
	*P. ornata*	–	0.94–0.97	0.32–0.85	0.52	–	–	[77, 179, 213]
	*P. streckeri*	–	–	–	–	1–3 years	–	[132, 214]
Crucifer	*P. crucifer*	0.52	0.5–0.9	0.25	0.25	4 years	2 years	[99, 168, 203, 215, 104, 215–221]
	*P. ocularis*	–	0.1	–	–	–	–	[89]
Trilling frog	*P. brachyphona*	–	–	–	–	–	–	–
	*P. brimleyi*	–	–	–	–	–	–	–
	*P. clarkii*	0.39	0.22–0.84	–	–	1–2 years	–	[204, 214, 222, 223]
	*P. feriarum*	0.77	0.10–0.89	–	–	–	–	[202, 224]
	*P. fouquettei*	–	–	–	–	–	–	–
	*P. kalmi*	–	–	–	–	–	–	–
	*P. maculata*	0.4–0.9	0.3–0.9	0.09–0.13	0.14–0.49	2–7 years	1 year	[99, 105, 128, 129, 181, 114, 203, 225, 226, 225–228]
	*P. nigrita*	–	–	–	0.28	1–3 years	–	[77]
	*P. triseriata*	0.37–0.87	0.9	0.06–0.13	0.19	1–3 years	1–2 years	[113, 77, 127, 205, 217]

To simplify the table, male and female survival parameters have been grouped

##### Juveniles

There is considerable uncertainty in survival probabilities of juvenile chorus frogs, a pattern that is observed for many amphibians [229, 230]. The complexity of marking and recapturing metamorphic and juvenile anurans make estimating survival very difficult [225]. For many chorus frog species, data are lacking. Studies that estimated juvenile survival probabilities in natural environments found that only a small proportion of froglets reach the adult stage. For example, in a study on *P. illoniensis,* Tucker [210] estimated a survival probability from metamorphosis to sexual maturity to be only 0.03. Whiting [105] estimated juvenile survival probability to be approximately 0.09–0.13 (*P. maculata*), whereas Smith [127] found survival probability of juveniles to adulthood was approximately 0.19 (*P. triseriata*). However, these three authors did not correct for imperfect detection probabilities, so actual survival could be very different from the reported estimates [231, 232]. Advancements in mark and recapture technology, such as small, light-weight visible implant elastomer tags [233, 234] and alpha tags [235] offer the possibility of improved juvenile population estimations.

##### Adults

We found survival estimates for seven (38%) of the 18 *Pseudacris* species. For these species, the probability of survival varied between 0.01 and 0.52. Studies on the same species report conflicting adult survival rates. For example, Muths et al. [128] estimated that mean adult survival probability in *P. maculata* was approximately 0.51 (both sexes combined), whereas survival estimates from Whiting [105] ranged from 0.25 to 0.27 in males and 0.36–0.50 in females. These discrepancies could be due to different analytical approaches: Muths et al. [128] used a formal capture-mark-recapture model, whereas Whiting [105] used an ad hoc estimate of survival that did not account for recapture probability. Most studies on *Pseudacris* are relatively short in duration, spanning only 2–3 years, and may not accurately capture variability of survival among years. Notable exceptions are the 30-year studies on two populations of *P. maculata* in Colorado, USA by Muths et al. [128] and Kissel, Tenan and Muths [129]. Between years, Muths et al. [128] observed highly variable survival probabilities ranging from 0.19 to 0.76. Therefore, studies on adult survival probability should extend several years to capture variation in environmental conditions (hydroperiod, temperature, predation) and their impact.

### Longevity and iteroparity

The majority of studies indicate that *Pseudacris* species have a lifespan between 1 and 3 years [66, 77, 105, 113, 236]. However, several studies suggest longevity in chorus frogs is underestimated. Using skeletochronology, Lykens and Forester [215] estimated that *P. crucifer* could live for 4 years (n = 3 individuals, out of 43 studied). Using capture-mark-recapture methods, Tucker et al. [211] reported that some adults of *P. illinoensis* reached 6 years (mean 2–3 years). Using a similar approach, Muths et al. [128, 226] recaptured tagged female *P. maculata* that were 7 years old. Together, this indicates that chorus frogs have a lifespan beyond the previously believed 1–3 years, but that individuals experience low survival between breeding seasons. Longevity estimates may be male-biased, as males are captured more easily during reproduction than females [128]. Conversely, if females occur close to a breeding site during several consecutive years, it may be assumed that females attempt breeding at least twice within their lifespan (Muths E., pers. com.).

### Conservation status

All 18 species in the genus of *Pseudacris* are currently classified as “least concern” by the International Union for the Conservation of Nature [2]. Global population trends are considered “stable” for the majority of species (Table 3). However, the IUCN states that population trends are unknown for *P. brachyphona*, *P. illinoensis*, and *P. streckeri*, and considered decreasing for *P. triseriata*. Currently, IUCN considers *P. hypochondriaca* and *P. sierra* as subspecies of *P. regilla*, and *P. illinoensis* as a subspecies of *P. streckeri*. It is possible that the rankings and population trends of these species could change if assessed separately. According to Recuero et al. [48], even if the three members of the *P. regilla* complex were considered separate species by the IUCN, they would still likely be classified as “least concern”. The patterns reported by the IUCN concur with the status designated by the governments of Canada, USA, and Mexico. All three species that occur in Mexico (*P. cadaverina, P. hypochondriaca, P. clarkii*) have a status of “least concern” despite observed declines and persistent threats to populations of *P. hypochondriaca* [237]. In Canada, the Great Lakes/St. Lawrence—Canadian Shield population of *P. maculata* (distributed in Ontario and Québec) is designated as threatened and is listed under the Species at Risk Act [238]. Sub-nationally in Québec, *P. maculata* is listed as vulnerable (high risk of extirpation) under the Act Respecting Threatened or Vulnerable Species (R.S.Q., c. E-12.01), as the species is estimated to occupy only 10% of its historical range [239, 240]. The population was previously designated as *P. triseriata* [241, 242], which may contribute to why the IUCN now considers the species populations to be declining. None of the species that occur in the USA are listed under the Endangered Species Act [243], but the status of *P. illinoensis* is currently under review by the US Fish and Wildlife Service [244]. According to NatureServe [117] databases, all species are “secure” at the national level, with the exception of *P. illinoensis* (N3 = Vulnerable), *P. kalmi* (N4 = Apparently Secure), and *P. cadaverina* (N4 = Apparently Secure). However, several populations are considered critically imperiled, or at a very high risk of being extirpated, at the subnational level, including *P. streckeri* (in Louisiana), *P. ocularis* (in Alabama), *P. brachyphona* (in Maryland), *P. brimleyi* (in Georgia), *P. feriarum* (in Pennsylvania), *P. kalmi* (in Pennsylvania), *P. maculata* (in Michigan and Vermont), and *P. triseriata* (in Pennsylvania). Populations of *P. illinoensis* in Illinois are classified as threatened [245] and the species has a very restricted distribution in Arkansas [236].

**Table 3:** Summary of the national and subnational status of frog species in the genus *Pseudacris*, separated by clade

Clade	Species	National distribution	IUCN Status	IUCN Trend	NatureServe subnational status rank (CAN & USA)
West coast	*P. cadaverina*	USA, MEX	Least concern	Stable	**CA**: SNR
	P. hypochondriaca*	USA, MEX	Least concern*	Stable*	**CA, NV**: SNR; **UT**: SU; **AZ**: S3
	P. sierra*	USA	Least concern*	Stable*	**CA, OR**: SNR; **UT**: SH; **MT**: S4; **ID, NV**: S5
	*P. regilla*	USA, CAN	Least concern	Stable	**AK, CA**: SNR; **MT**: S4; **BC, OR, WA**: S5
Fat frog	*P. illinoensis*^†^	USA	Least concern^†^	Unknown^†^	**AR**: S1; **MO**: S2; **IL**: S2S3
	*P. ornata*	USA	Least concern	Stable	**LA**: SH; **MS**: S1; **NC**: S2; **FL**: S2S3; **SC**: S3S4; **AL, GA**: S5
	*P. streckeri*	USA	Least concern	Unknown	**IL, MO, OK**: SNR; **LA**: S1; **AR, KS**: S2; **TX**: S3
Crucifer	*P. crucifer*	USA, CAN	Least concern	Stable	**FL, IN, OH, OK, SC**: SNR; **NL**: S1S2; **KS**: S3; **DC, IA, MN**: S4; **MB, NB, NS, ON, PE, QC, AL, AR, CT, DE, GA, IL, KY, LA, ME, MD, MI, MS, MO, NH, NJ, NY, NC, PA, RI, TN, TX, VT, VA, WV, WI**: S5
	*P. ocularis*	USA	Least concern	Stable	**SC**: SU; **FL**: SNR; **AL**: S1; **VA**: S3; **GA**: S4S5; **NC**: S5
Trilling frog	*P. brachyphona*	USA	Least concern	Unknown	**OH**: SNR; **MD**: S1; **GA, NC, PA**: S2; **MS**: S3; **TN, VA, WV**: S4; **KY**: S5
	*P. brimleyi*	USA	Least concern	Stable	**SC**: SNR; **GA**: S1; **NC, VA**: S4
	*P. clarkii*	USA, MEX	Least concern	Stable	**OK**: SNR; **KS, TX**: S5
	*P. feriarum*	USA	Least concern	Stable	**NJ**: SU; **FL**: SNR; **PA**: S1; **DC, WV**: S3; **IL**: S4; **AL, GA, KY, MD, MS, MO, NC, SC, TN, VA**: S5
	*P. fouquettei*	USA	Least concern	Stable	**TX**: SU; **MS, MO**: SNR; **OK**: S3; **AR, LA**: S5
	*P. kalmi*	USA	Least concern	Stable	**VA**: SNR; **PA**: S1; **NJ**: S3; **DE, MD**: S4
	*P. maculata*	USA, CAN	Least concern	Stable	**ND, OK**: SNR; **MI, VT**: S1; **YT**: S1S2; **QC, IN**: S2; **NY**: S2S3; **NM**: S3; **ID, IA, ON, UT**: S4; **BC, NT**: S4S5; **AB, MB, ON, SA, AZ, CO, IL, KS, MN, MO, MT, NE, SD, WI, WY**: S5
	*P. nigrita*	USA	Least concern	Stable	**FL, LA, SC**: SNR; **NC**: S2; **VA**: S3; **AL, GA, MS**: S5
	*P. triseriata*	USA, CAN	Least concern	Decreasing	**QC, IL, OH**: SNR; **PA**: S1; **NY**: S2S3; **ON, IN**: S4; **KY, MI**: S5

NatureServe subnational ranks range from most at risk of extinction (critically imperilled; S1) to least at risk of extinction (stable; S5), and include ranks for species that are unrankable (SU), currently unranked (SNR), or presumed to be extirpated (SH). Multiple ranks combined (i.e., S2S3) indicate uncertainty of conservation status. See Additional files 3, 4: Table S3–S4 for a more detailed descriptions of ranking and a full list of abbreviations. Region abbreviation (i.e., CA, NV, UT, etc.) are bolded for legibility and hold no other significance

^*^Species considered a subspecies of *P. regilla* by IUCN

^†^Species considered a subspecies of *P. streckeri* by IUCN

## Conclusion

The current state of knowledge on the ecology, life history strategies and conservation status of North American chorus frogs has been reviewed. We found that the life history traits of *Pseudacris* are largely consistent among species, which is noteworthy considering their relative diversity and wide distribution in North America. Among an equally widespread clade, the 22 species of American water frogs (genus *Lithobates*: Ranidae), there is comparatively more variation in life histories [50, 57]. For example, bullfrogs (*Lithobates catesbeiana*) are long-lived (8–10 years), have a prolonged breeding season from late spring to early summer, lay up to 20,000 eggs/clutch in permanent waterbodies with older females producing 2 clutches per year, and have a long larval period of up to 3 years [50]. In comparison, wood frogs (*Lithobates sylvatica*) live approximately 4–5 years, have an “explosive” breeding strategy where the majority of individual breed shortly after the first rainfall in late winter, lay 300–1500 eggs in a communal location within semi-permanent ponds, and have a relatively short larval period of 65–130 days [50]. Finally, carpenter frogs (*Lithobates virgatipes*) live 3–4 years, have a prolonged breeding period from May to July, lay only 200–600 eggs in permanent wetlands, and have a larval period of approximately 1 year [50].

Whereas no major differences in life history traits emerge among *Pseudacris* species, the distribution of the populations appears to impact clutch size and development. Within a species, females in warmer, more southern populations tend to have smaller clutches of eggs, but the extended breeding season allows for multiple clutches within a single season. Therefore, total annual egg production amongst species is very similar. Eggs of populations in warmer climates also hatch sooner and develop more quickly than those in more temperate climates. No clear associations between conservation status and life history strategies were detected. While many populations of species within the Trilling Frog clade are considered critically imperilled at the subnational level (Table 3), we speculate that this is likely a result of restricted distribution and increased local threats in distal portions of the species’ range. The majority of the populations in decline occur in the east coast of the USA. For example, three species (*P. feriarum*, *P. kalmi*, and *P. triseriata*) are at a high level of risk of extinction in Pennsylvania. The factors contributing to this extinction risk in Pennsylvania may be related to the spread of disease, the high human population density (9th highest of the 50 US states), and increased levels of anthropogenic disturbance [246], but this should be investigated extensively.

The most striking finding in our review is the scarcity of data on the egg, larval, and juvenile life stages. For many species there are no data available on the number of eggs laid or the length of the embryonic period, particularly in the West Coast and Fat Frog clades (see *P. cadaverina* and *P. illinoensis* in Table 1). Data are lacking for estimates of stage-specific survival rates and longevity for most chorus frog species (Table 2). More than two-thirds of data that have been collected prior to the 2000s, highlighting the need for a reassessment addressing the recent updates to phylogeny [43, 44, 52, 53]. The focus should shift to species that have been historically underrepresented or have been conflated with other species, including those recently elevated to species status (i.e., *P. fouquettei, P. hypochondriaca, P. illinoensis, P. sierra*).

Information on life history traits is critical for understanding the ecology of chorus frogs and will improve our understanding of how environmental threats impact populations. Empirical data are also required for species conservation and mitigation efforts, to prevent further declines in regions where populations appear relatively stable or unaffected, maintaining common species common [247]. We have found that there are many similarities in life history traits among species in the *Pseudacris* genus. Chorus frogs may therefore be generally susceptible to the same anthropogenic disturbance and changing climate patterns due to their characteristic cold weather breeding strategy and reliance on temporary wetlands. More promising is that the strong similarities in life histories and reproductive ecology of the 18 identified *Pseudacris* species suggests that recovery strategies we can develop for one species could be more broadly applicable. Thus, the data collected on species (or populations) that are currently stable can inform and benefit conservation efforts on species and populations declining elsewhere in North America. There is great potential for meaningful and impactful collaboration among research and conservation groups throughout the continent.

## Supplementary Information

**Additional file 1**. **Table S1**. List of official common names and scientific names of chorus frogs (genus Pseudacris: Hylidae), Society for the Study of Amphibians and Reptiles 57.

**Additional file 2**. **Table S2**. List of key words used in Google Scholar and Web of Science databases to retrieve articles on life history traits, survival rates, and longevity of Pseudacris species in North America.

**Additional file 3**. **Table S3**. List of abbreviations of the countries, and their provinces, states and territories of North America.

**Additional file 4**. **Table S4**. Explanation of the NatureServe status rank codes.

## Data Availability

The datasets used and analysed during the current study are available from the corresponding author on reasonable request.
