# Supplementary material for: Life history traits and reproductive ecology of North American chorus frogs of the genus Pseudacris (Hylidae)
Source: Front Zool. 2021 Aug 27;18:40. doi: 10.1186/s12983-021-00425-w (PMC8394169; doi:10.1186/s12983-021-00425-w)
Supplement: Supplementary file 1 — Additional file 1. Table S1. List of official common names and scientific names of chorus frogs (genus Pseudacris: Hylidae), Society for the Study of Amphibians and Reptiles 57. [file 12983_2021_425_MOESM1_ESM.docx]

**Table S1:** List of official common names and scientific names of chorus frogs (genus *Pseudacris:* Hylidae), Society for the Study of Amphibians and Reptiles (57)

| **Common Name** | **Scientific Name** |
| --- | --- |
| Mountain Chorus Frog | *Pseudacris brachyphona* (Cope, 1889) |
| Brimley's Chorus Frog | *Pseudacris brimleyi* (Brandt and Walker, 1933) |
| California Chorus Frog | *Pseudacris cadaverina* (Cope, 1866) |
| Spotted Chorus Frog | *Pseudacris clarkii* (Baird, 1854) |
| Collinses’ Mountain Chorus Frog | *Pseudacris collinsorum* (Ospina, Tieu, Apodaca, and Lemmon, 2020) |
| Spring Peeper | *Pseudacris crucifer* (Wied-Neuwied, 1838) |
| Upland Chorus Frog | *Pseudacris feriarum* (Baird, 1854) |
| Cajun Chorus Frog | *Pseudacris fouquettei* (Lemmon, Lemmon, Collins, and Cannatella, 2008) |
| Baja California Treefrog | *Pseudacris hypochondriaca* (Hallowell, 1854) |
| Illinois Chorus Frog | *Pseudacris illinoensis* (Smith, 1951) |
| New Jersey Chorus Frog | *Pseudacris kalmi* (Harper, 1955) |
| Boreal Chorus Frog | *Pseudacris maculate* (Agassiz, 1850) |
| Southern Chorus Frog | *Pseudacris nigrita* (LeConte, 1825) |
| Little Grass Frog | *Pseudacris ocularis* (Holbrook, 1838) |
| Ornate Chorus Frog | *Pseudacris ornate* (Holbrook, 1836) |
| Northern Pacific Treefrog | *Pseudacris regilla* (Baird and Girard, 1852) |
| Sierran Treefrog | *Pseudacris sierra* (Jameson, Mackey, and Richmond, 1966) |
| Strecker's Chorus Frog | *Pseudacris streckeri* (Wright and Wright, 1933) |
| Western Chorus Frog | *Pseudacris triseriata* (Wied-Neuwied, 1838) |
