# Supplementary material for: Life history traits and reproductive ecology of North American chorus frogs of the genus Pseudacris (Hylidae)
Source: Front Zool. 2021 Aug 27;18:40. doi: 10.1186/s12983-021-00425-w (PMC8394169; doi:10.1186/s12983-021-00425-w)
Supplement: Supplementary file 2 — Additional file 2. Table S2. List of key words used in Google Scholar and Web of Science databases to retrieve articles on life history traits, survival rates, and longevity of Pseudacris species in North America. [file 12983_2021_425_MOESM2_ESM.docx]

**Table S2:** List of key words used in Google Scholar and Web of Science databases to retrieve articles on life history traits, survival rates, and longevity of *Pseudacris* species in North America. For Web of Science searches, “TS” is used to search terms within article topics and “TI” is used to search for terms in article titles.

| **Database** | **Acronym Used** |
| --- | --- |
| **Web of Science** | TS=(chorus frog* OR Pseudacris OR Trilling frog*) AND TS=(survival OR survivorship OR fertility OR demography OR longevity OR lifespan OR dynamic OR growth OR reproductive success OR life history) |
|  | TI=(chorus frog* OR Pseudacris OR Trilling frog*) AND TS= (survival OR survivorship OR recruitment* OR population size OR demography OR longevity OR lifespan OR population dynamic OR growth rate OR reproductive success OR life history) |
|  | TI=(chorus frog* OR Pseudacris OR Trilling frog*) AND TI=(survival OR survivorship OR fertility OR demography OR longevity OR lifespan OR dynamic OR growth OR reproductive success OR life history) |
|  | TI=(chorus frog* OR Pseudacris maculata OR boreal chorus frog OR Pseudacris triseriata OR Pseudacris crucifer) AND TI =(survival OR survivorship OR fertility OR demography OR longevity OR lifespan OR dynamic OR growth OR reproductive success) |
|  | TS=(chorus frog* OR Pseudacris maculata OR boreal chorus frog OR Pseudacris triseriata OR Pseudacris crucifer) AND TI =(survival OR survivorship OR fertility OR demography OR longevity OR lifespan OR dynamic OR growth OR reproductive success) |
|  | TI=(chorus frog* OR Pseudacris maculata OR boreal chorus frog OR Pseudacris triseriata OR Pseudacris crucifer) AND TI=(survival OR survivorship OR fertility OR demography OR longevity OR lifespan OR dynamic OR growth OR reproductive success OR life history) |
|  | TS=(egg OR larvae OR tadpole OR clutch OR life history) AND TS=(chorus frog OR Pseudacris) |
|  | TS=(eggs OR larvae OR tadpole OR fertility OR oviposition OR life-history) AND TI=(chorus frog OR Pseudacris) |
| **Google Scholar** | Survival OR survivorship OR fertility OR demography OR longevity OR lifespan OR dynamic OR growth OR reproductive OR success OR life history "chorus frog* OR Pseudacris OR Trilling frog*” |
|  | (chorus frog* OR Pseudacris maculata OR boreal chorus frog OR Pseudacris triseriata OR Pseudacris crucifer) AND (survival OR survivorship OR fertility OR demography OR longevity OR lifespan OR dynamic OR growth OR reproductive success OR life history) |
|  | (chorus frog* OR Pseudacris maculata OR boreal chorus frog OR Pseudacris triseriata OR Pseudacris crucifer) AND (survival OR survivorship OR fertility OR demography OR longevity OR lifespan OR dynamic OR growth OR reproductive success) |
|  | (egg* OR larva* OR tadpole OR clutch OR reproductive OR life history) AND ("chorus frog*" OR Pseudacris OR "Trilling frog*") |
|  | Pseudacris eggs OR larva OR tadpole OR fertility OR oviposition OR "life history" "chorus frog" |
|  | ("Hyla cadaverina") AND (clutch OR "life history" OR reproduction OR oviposition OR development) |
|  | ("Hyla californiae") AND (clutch OR "life history" OR reproduction OR oviposition OR development) |
|  | ("Hyla crucifer") AND (clutch OR "life history" OR reproduction OR oviposition OR development) |
|  | ("Hyla ocularis") AND (clutch OR "life history" OR reproduction OR oviposition OR development) |
|  | ("Hyla regilla") AND (clutch OR "life history" OR reproduction OR oviposition OR development) |
|  | ("Hyliola cadaverina") AND (clutch OR "life history" OR reproduction OR oviposition OR development) |
|  | ("Hyliola regilla") AND (clutch OR "life history" OR reproduction OR oviposition OR development) |
