# Supplementary material for: Life history traits and reproductive ecology of North American chorus frogs of the genus Pseudacris (Hylidae)
Source: Front Zool. 2021 Aug 27;18:40. doi: 10.1186/s12983-021-00425-w (PMC8394169; doi:10.1186/s12983-021-00425-w)
Supplement: Supplementary file 3 — Additional file 3. Table S3. List of abbreviations of the countries, and their provinces, states and territories of North America. [file 12983_2021_425_MOESM3_ESM.docx]

**Table S3:** List of abbreviations of the countries, and their provinces, states and territories of North America using the 2-letter state/province/territory codes for USA and Canada and the 3-letter state codes for Mexico (ISO 3166-2)

| **Country** | **State/Province/Territory** | **Code** |
| --- | --- | --- |
| Canada (CAN) | Alberta | AB |
|  | British Columbia | BC |
|  | Manitoba | MB |
|  | New Brunswick | NB |
|  | Newfoundland and Labrador | NL |
|  | Northwest Territories | NT |
|  | Nova Scotia | NS |
|  | Nunavut | NU |
|  | Ontario | ON |
|  | Prince Edward Island | PE |
|  | Quebec | QC |
|  | Saskatchewan | SK |
|  | Yukon | YT |
| United States of America (USA) | Alabama | AL |
|  | Alaska | AK |
|  | Arizona | AZ |
|  | Arkansas | AR |
|  | California | CA |
|  | Colorado | CO |
|  | Connecticut | CT |
|  | Delaware | DE |
|  | District of Columbia | DC |
|  | Florida | FL |
|  | Georgia | GA |
|  | Hawaii | HI |
|  | Idaho | ID |
|  | Illinois | IL |
|  | Indiana | IN |
|  | Iowa | IA |
|  | Kansas | KS |
|  | Kentucky | KY |
|  | Louisiana | LA |
|  | Maine | ME |
|  | Maryland | MD |
|  | Massachusetts | MA |
|  | Michigan | MI |
|  | Minnesota | MN |
|  | Mississippi | MS |
|  | Missouri | MO |

**Table S3 (continued):** List of abbreviations of the countries, and their provinces, states and territories of North America using the 2-letter state/province/territory codes for USA and Canada and the 3-letter state codes for Mexico (ISO 3166-2)

| **Country** | **State/Province/Territory** | **Code** |
| --- | --- | --- |
| United States of America (USA) | Montana | MT |
|  | Nebraska | NE |
|  | Nevada | NV |
|  | New Hampshire | NH |
|  | New Jersey | NJ |
|  | New Mexico | NM |
|  | New York | NY |
|  | North Carolina | NC |
|  | North Dakota | ND |
|  | Ohio | OH |
|  | Oklahoma | OK |
|  | Oregon | OR |
|  | Pennsylvania | PA |
|  | Rhode Island | RI |
|  | South Carolina | SC |
|  | South Dakota | SD |
|  | Tennessee | TN |
|  | Texas | TX |
|  | Utah | UT |
|  | Vermont | VT |
|  | Virginia | VA |
|  | Washington | WA |
|  | West Virginia | WV |
|  | Wisconsin | WI |
|  | Wyoming | WY |
| Mexico (MEX) | Aguascalientes | AGU |
|  | Baja California | BCN |
|  | Baja California Sur | BCS |
|  | Campeche | CAM |
|  | Chiapas | CHP |
|  | Chihuahua | CHH |
|  | Coahuila | COA |
|  | Colima | COL |
|  | Mexico City | CMX |
|  | Durango | DUR |
|  | Guanajuato | GUA |
|  | Guerrero | GRO |
|  | Hidalgo | HID |
|  | Jalisco | JAL |
|  | México | MEX |

**Table S3 (continued):** List of abbreviations of the countries, and their provinces, states and territories of North America using the 2-letter state/province/territory codes for USA and Canada and the 3-letter state codes for Mexico (ISO 3166-2)

| **Country** | **State/Province/Territory** | **Code** |
| --- | --- | --- |
| Mexico (MEX) | Michoacán | MIC |
|  | Morelos | MOR |
|  | Nayarit | NAY |
|  | Nuevo León | NLE |
|  | Oaxaca | OAX |
|  | Puebla | PUE |
|  | Querétaro | QUE |
|  | Quintana Roo | ROO |
|  | San Luis Potosí | SLP |
|  | Sinaloa | SIN |
|  | Sonora | SON |
|  | Tabasco | TAB |
|  | Tamaulipas | TAM |
|  | Tlaxcala | TLA |
|  | Veracruz | VER |
|  | Yucatán | YUC |
|  | Zacatecas | ZAC |
