# Supplementary material for: Life history traits and reproductive ecology of North American chorus frogs of the genus Pseudacris (Hylidae)
Source: Front Zool. 2021 Aug 27;18:40. doi: 10.1186/s12983-021-00425-w (PMC8394169; doi:10.1186/s12983-021-00425-w)
Supplement: Supplementary file 4 — Additional file 4. Table S4. Explanation of the NatureServe status rank codes. [file 12983_2021_425_MOESM4_ESM.docx]

**S4:** Explanation of the NatureServe status rank codes

NatureServes status ranks are based on species rarity, severity of threats, and population trends. These three broad categories are individually scaled and weighted based on their overall impact on the risk of extirpation. The scores for each category are combined to give an overall ranking, which is reviewed and then accepted (NatureServe 2021). As an example, we use the subnational (S) prefix.

SX: Presumed Extirpated – no evidence of species being extant despite extensive searches and very low likelihood of the species being rediscovered.

SH: Possibly Extirpated – historical records exist but has not been recently documented (20-40 years) but there is uncertainty about this assessment as thorough searches have not been performed.

S1: Critically Imperiled – very high risk of extirpation due to restricted range, low abundance, recent and widespread declines, threats, or other factors.

S2: Imperiled – high risk of extirpation due to restricted range, low abundance, recent and widespread declines, threats, or other factors.

S3: Vulnerable – moderate risk of extirpation due to restricted range, low abundance, recent and widespread declines, threats, or other factors.

S4: Apparently Secure – relatively low risk of extirpation but certain populations may be a risk of local declines, threats, or other factors.

S5: Secure – very low or no risk of extirpation.

SNR: Unranked – status not yet assessed.

SU: Unrankable – unable to rank due to a lack of information or due to conflicting information.

S#S#: Range Rank – used to indicate uncertainty
